# Identification of deferasirox as a human xanthine oxidase inhibitor

**DOI:** 10.1093/lifemedi/lnaf014

**Published:** 2025-03-31

**Authors:** Yunfei Qi, Xinheng He, Xiaoshan Wu, Tingting Zhou, Ningning Liang, Jiazheng Jiao, Yanhao Chen, Yue Yuan, Yuwei Zhang, Yucheng Wang, Yan Liu, Qiurong Ding

**Affiliations:** CAS Key Laboratory of Nutrition, Metabolism and Food Safety, Shanghai Institute of Nutrition and Health, Shanghai Institutes for Biological Sciences, University of Chinese Academy of Sciences, Chinese Academy of Sciences, Shanghai 200031, China; CAS Key Laboratory of Receptor Research, Shanghai Institute of Materia Medica, University of Chinese Academy of Sciences, Chinese Academy of Sciences, Shanghai 201203, China; CAS Key Laboratory of Nutrition, Metabolism and Food Safety, Shanghai Institute of Nutrition and Health, Shanghai Institutes for Biological Sciences, University of Chinese Academy of Sciences, Chinese Academy of Sciences, Shanghai 200031, China; CAS Key Laboratory of Nutrition, Metabolism and Food Safety, Shanghai Institute of Nutrition and Health, Shanghai Institutes for Biological Sciences, University of Chinese Academy of Sciences, Chinese Academy of Sciences, Shanghai 200031, China; CAS Key Laboratory of Nutrition, Metabolism and Food Safety, Shanghai Institute of Nutrition and Health, Shanghai Institutes for Biological Sciences, University of Chinese Academy of Sciences, Chinese Academy of Sciences, Shanghai 200031, China; CAS Key Laboratory of Nutrition, Metabolism and Food Safety, Shanghai Institute of Nutrition and Health, Shanghai Institutes for Biological Sciences, University of Chinese Academy of Sciences, Chinese Academy of Sciences, Shanghai 200031, China; CAS Key Laboratory of Nutrition, Metabolism and Food Safety, Shanghai Institute of Nutrition and Health, Shanghai Institutes for Biological Sciences, University of Chinese Academy of Sciences, Chinese Academy of Sciences, Shanghai 200031, China; CAS Key Laboratory of Nutrition, Metabolism and Food Safety, Shanghai Institute of Nutrition and Health, Shanghai Institutes for Biological Sciences, University of Chinese Academy of Sciences, Chinese Academy of Sciences, Shanghai 200031, China; CAS Key Laboratory of Nutrition, Metabolism and Food Safety, Shanghai Institute of Nutrition and Health, Shanghai Institutes for Biological Sciences, University of Chinese Academy of Sciences, Chinese Academy of Sciences, Shanghai 200031, China; CAS Key Laboratory of Nutrition, Metabolism and Food Safety, Shanghai Institute of Nutrition and Health, Shanghai Institutes for Biological Sciences, University of Chinese Academy of Sciences, Chinese Academy of Sciences, Shanghai 200031, China; CAS Key Laboratory of Nutrition, Metabolism and Food Safety, Shanghai Institute of Nutrition and Health, Shanghai Institutes for Biological Sciences, University of Chinese Academy of Sciences, Chinese Academy of Sciences, Shanghai 200031, China; CAS Key Laboratory of Nutrition, Metabolism and Food Safety, Shanghai Institute of Nutrition and Health, Shanghai Institutes for Biological Sciences, University of Chinese Academy of Sciences, Chinese Academy of Sciences, Shanghai 200031, China

## Dear editor,

The incidence of hyperuricemia, characterized by elevated serum uric acid (UA) concentration in extracellular fluid and tissues or impaired UA excretion, is on the rise globally due to the increasing prevalence of Western diets and lifestyle changes. Hyperuricemia can lead to gout and has been linked to the development of metabolic syndrome, type 2 diabetes, and cardiovascular diseases [1]. Xanthine oxidase (XO) catalyzes the conversion of hypoxanthine to UA via xanthine as an intermediate. XO inhibitors are currently used in clinical practice to treat gout or hyperuricemia [2]. However, the existing XO inhibitors (allopurinol, febuxostat, and topiroxostat) may exhibit side effects that can be serious in some cases [2], highlighting the urgent need for the development of novel, potent, and safer XO inhibitors globally.

**Figure 1.**
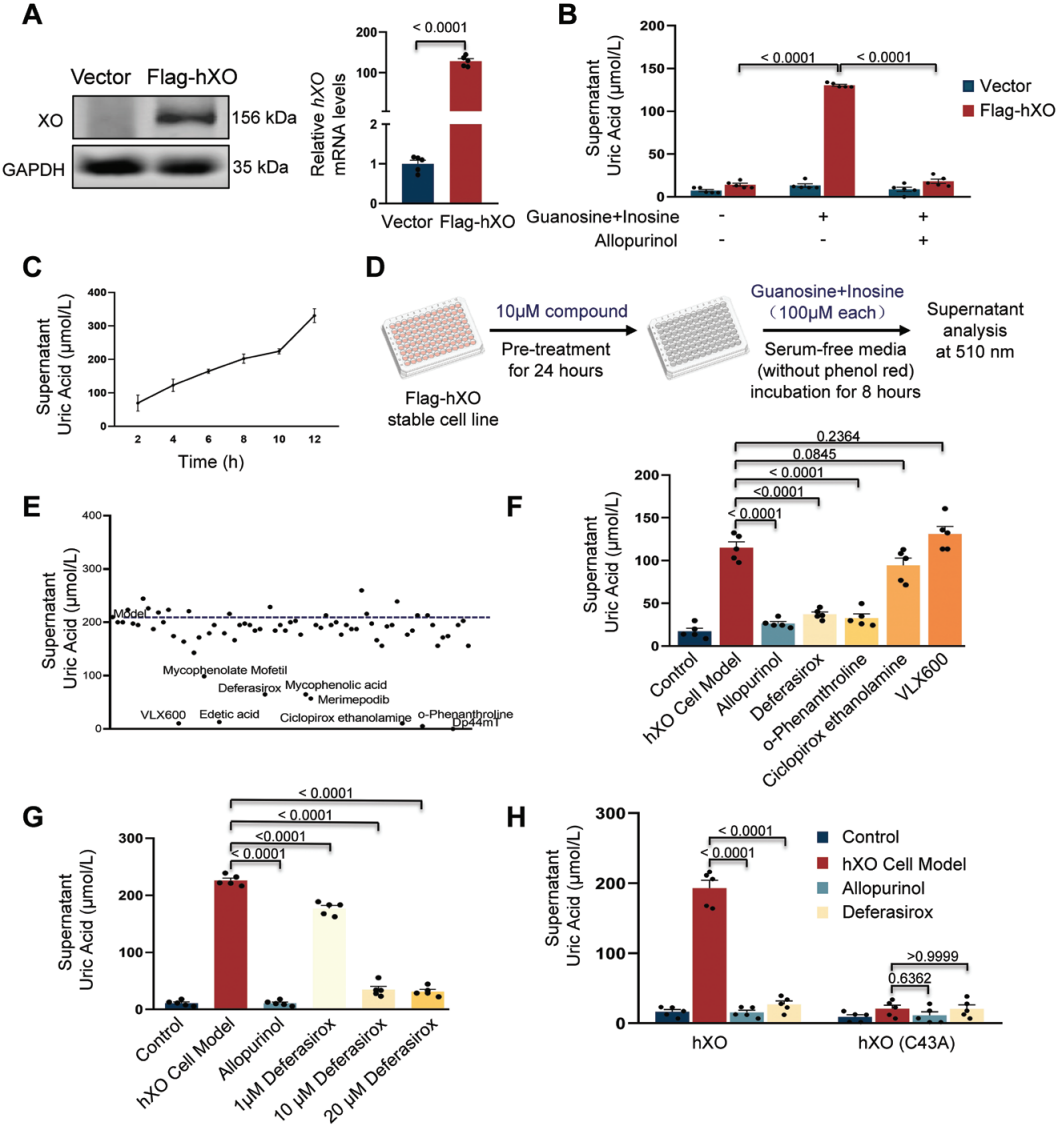
**High-throughput screening identifies deferasirox as an effective human xanthine oxidase inhibitor.**(A) Protein and mRNA analysis of human XO expression in a stable cell line (Flag-hXO) as compared to control cells (Vector) (*n* = 5). (B) Analysis of supernatant UA levels in cells after treatment of substrates (100 μM each) with or without the addition of allopurinol (1 μM) for 8 h (*n* = 5). (C) Analysis of supernatant UA levels in Flag-hXO cells after exposure to substrates (guanosine and inosine, 100 μM each) for different times, as indicated. *n* = 5. (D) Scheme illustration of the compound screening procedure. (E) Supernatant UA analysis after treatment of different compounds (10 μM) in the screening. The dashed line suggests UA levels in hXO cell model. Candidate drugs were chosen with > 30% reduction in UA levels when compared to levels in the hXO cell model. (F) Analysis of supernatant UA levels after treatment with individual compound at a concentration of 10 μM, as indicated. Control, cells with no compound treatment and no substrates supplementation containing DMSO; hXO cell model, cells without compounds pre-treatment 24 h and supplementation of substrates to activate UA production. *n *= 5. (G) Analysis of supernatant UA levels after treatment with deferasirox at different concentrations, as indicated. Allopurinol (1 μM) was applied as a positive control. *n* = 5. (H) Analysis of supernatant UA levels in stable cell lines expression exogenous hXO or hXO (C43A) after treatment with deferasirox (10 μM) or allopurinol (1 μM). *n* = 5. Values present means with s.e.m. *P* values were assessed by the unpaired, two-tailed Student’s *t* test (A), the one-way ANOVA (C, F and G) or the two-way ANOVA (B, H).

To enable high-throughput screening of human XO inhibitors, we established a stable mouse AML12 liver cell line expressing human XO through lentiviral delivery (Fig. 1A). The addition of guanosine and inosine substrates significantly enhanced UA production, which could be inhibited by treatment with the XO inhibitor allopurinol as a positive control (Fig. 1B). Furthermore, the UA production in this cell line exhibited a linear relationship with the exposure time to the substrates (Fig. 1C), and was dose-dependent on the applied allopurinol (Fig. S1A). These results collectively demonstrate the viability of this stable cellular model for the screening of XO inhibitors. Subsequently, we conducted the screening in 96-well plates, wherein cells were pre-treated with 10 μM individual compounds for 24 h, followed by exposure to substrates for an additional 8 h, and subsequent analysis of UA levels in medium (Fig. 1D).

XO is an Fe–S enzyme that exists as a homodimer with a molecular mass of 290 kDa. Each monomer contains two non-identical [2Fe–2S] centers, one molybdopterin group, and one flavin adenine dinucleotide (FAD) cofactor. The Fe–S centers play a critical role in electron transfer from molybdenum to FAD, thereby influencing the enzyme’s activity [3]. Based on this information, we hypothesized that metal chelators might interfere with the function of the Fe–S centers or the molybdenum in XO. To test this hypothesis, we collected 70 compounds known for their metal chelating activity (Table S1) and used them for screening (Figs. 1E and S1B). After further validation of UA-lowering effects and cell viability tests, deferasirox emerged as an intriguing candidate, exhibiting a dose-dependent inhibitory effect on UA production without significant toxicity to cells (Figs. 1F, 1G, S1C and S1D).

Deferasirox, an iron chelator, is the first oral medication approved by FDA for chronic iron overload in patients receiving long term blood transfusions. The inhibitory effect of deferasirox on human XO may be attributed to its iron chelation properties, which warrants further investigation. It is worth noting that Cys43 in the Fe–S centers serves as a ligand for one of the iron atoms [4] (Fig. S1E), and mutation of this Cys43 to alanine resulted in reduced enzyme activity (Fig. 1H), thereby highlighting the critical role of the Fe–S center in enzyme activity. Deferasirox has no further inhibitory effect on the hXO (C43A) mutant (Fig. 1H). Additionally, both allopurinol and deferasirox treatments led to decreased expression of several enzymes involved in purine metabolism, suggesting some feedback effects resulting from reduced UA production (Fig. S1F and S1G).

We then proceeded to investigate the UA-lowering effect of deferasirox in an *in vivo* setting. The uricase produced in the livers of rodents is capable of further breaking down UA into allantoin, thereby impeding the creation of appropriate rodent models for hyperuricemia. To achieve this, we employed a hyperuricemia animal model by targeting the *Uox* gene (which encodes urate oxidase) in liver cells using CRISPR/Cas9 technology [5], aiming to mimic human purine metabolism. Additionally, the animals were treated with hypoxanthine for a consecutive period of 7 days (Fig. S2A and S2B). The combination of liver *Uox* depletion and hypoxanthine treatment resulted in a significant increase in serum UA levels, as well as elevated levels of serum creatinine and blood urea nitrogen (Fig. S2C). Consequently, we referred to the animals in this group (*Uox* sgRNA + hypoxanthine) as the mHUA (mouse hyperuricemia) model.

**Figure 2.**
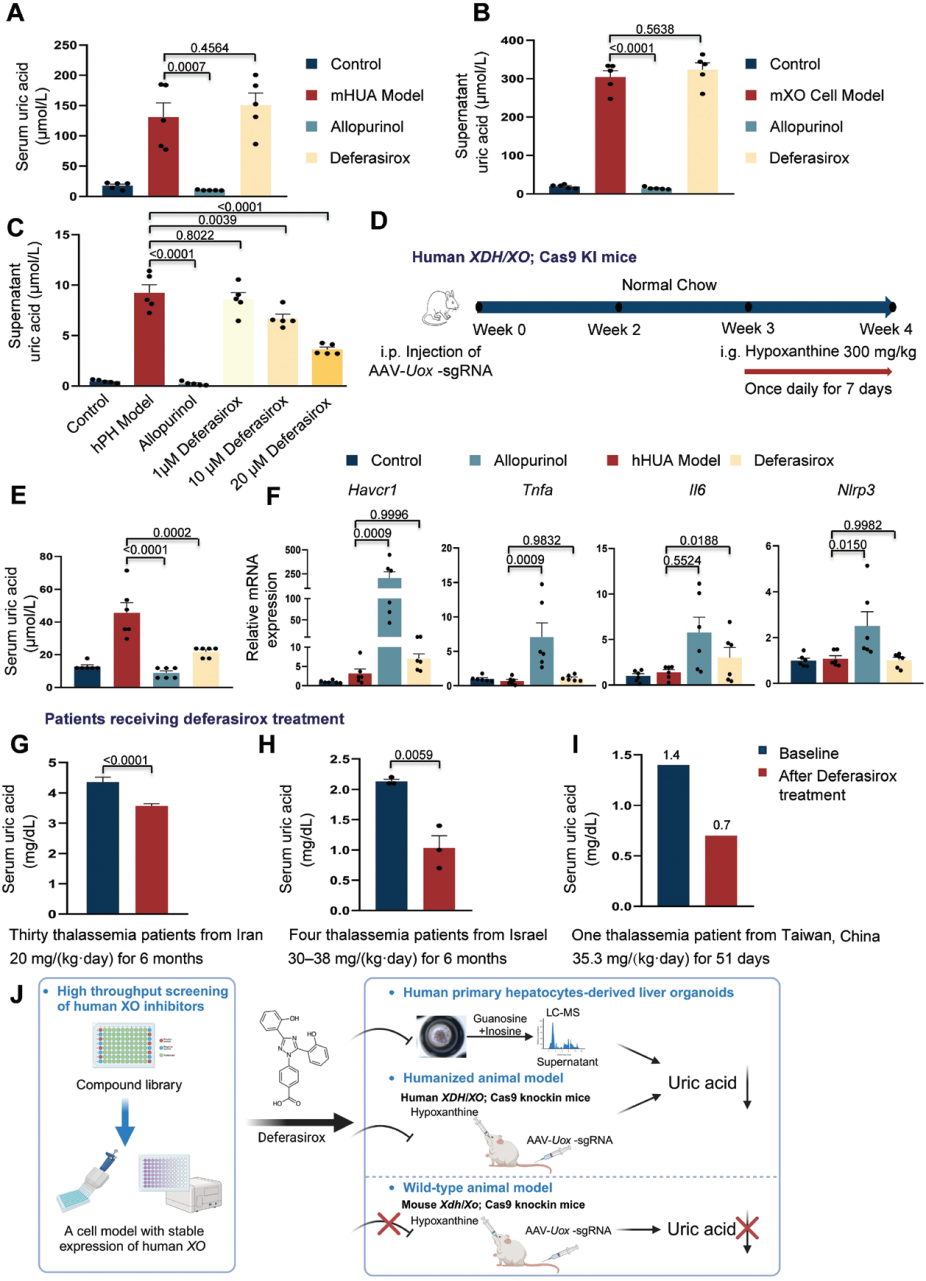
**Deferasirox treatment reduces UA production in a humanized hyperuricemia animal model.**(A) Animals injected for liver *Uox* depletion after 3 weeks were randomly divided into four groups (*n* = 5). Analysis of serum UA levels in a mouse hyperuricemia model (mHUA model) following oral administration of allopurinol (20 mg/kg) or deferasirox (20 mg/kg), as indicated. Control, animals were orally administrated with 0.5% CMC-Na solution. (B) Analysis of supernatant UA levels in a stable cell line expression exogenous mXO (mXO cell model) after treatment with deferasirox (10 μM) or allopurinol (1 μM). *n* = 5. (C) Analysis of supernatant UA levels in hPH Models after treatment with deferasirox at different concentrations, as indicated. Allopurinol (1 μM) was applied as a positive control. *n* = 5. (D) Scheme illustration of establishing a hyperuricemia animal model using hXO-humanized animals. (E) Animals injected for liver *Uox* depletion after 3 weeks were randomly divided into four groups (*n* = 6). Analysis of serum UA levels in the humanized hyperuricemia animal model (hHUA model) after treatment of allopurinol (20 mg/kg) or deferasirox (20 mg/kg), as indicated.Control, animals were orally administrated with 0.5% CMC-Na solution. (F) Analysis of gene expression levels in kidney tissues in animals. *n* = 6. (G–I) Analysis of patients' serum UA levels before and after treatment of deferasirox from three different studies. (J) Graphical summary. This cartoon was created by Biorender. Values present means with s.e.m. *P* values were assessed by the one-way ANOVA (A, B, C, E and F), or the unpaired, two-tailed Student's *t* test (G, H).

Interestingly, when we administrated deferasirox to these animals, we observed no reduction in serum UA levels (Fig. 2A). To further confirm this result, we utilized an AML12 cell line expressing mouse XO and supplemented it with substrates. Deferasirox treatment did not affect UA production in the cell line (Fig. 2B), thus confirming the lack of inhibitory effect of deferasirox on mouse XO activity.

To validate the inhibitory effect of deferasirox on human XO, we generated liver organoids derived from human primary hepatocytes [6] and treated these cells with deferasirox. We observed a dose-dependent reduction in UA production without significant toxicity to cells in human liver organoids upon deferasirox treatment (Figs. 2C and S2D), confirming its ability to suppress human XO activity.

To further corroborate the inhibitory function of deferasirox on human XO in an *in vivo* context, we generated a humanized mouse model by replacing the mouse *XO/XDH* gene with its human counterpart (Fig. S2E). These humanized mice were then subjected to *Uox* targeting and hypoxanthine treatment, resulting in the hHUA (human hyperuricemia) model (Fig. 2D). Notably, although the serum UA levels in the hHUA model were significantly increased compared to Control (Fig. 2E), they remained substantially lower than those observed in the mHUA model (Fig. 2A), likely due to the lower expression of human XO in the humanized animals compared to mouse XO expression in wild-type animals (Fig. S2F). Moreover, no significant changes in serum creatinine or blood urea nitrogen levels were observed in the hHUA model due to relatively low UA levels (Fig. S2G). Nonetheless, we observed a significant inhibitory effect of deferasirox on UA production in the hHUA model (Fig. 2E).

Interestingly, qPCR analysis revealed that allopurinol treatment induced the expression of several inflammation markers, such as *Havcr1* (encoding kidney injury molecule1, KIM-1), *Tnfa* (encoding tumor necrosis factor alpha), *Il6* (encoding interleukine 6), and *Nlrp3* (encoding NLR family pyrin domain containing 3). This suggests an increase in inflammatory signals upon allopurinol treatment (Fig. 2F). In contrast, deferasirox treatment did not significantly alter the expression levels of these genes (Fig. 2F), indicating that deferasirox might be a safer XO inhibitor for short-term treatment.

To further support our findings, we next retrieved information from three different studies involving patients who received deferasirox treatment for chronic iron overload [7–9]. Consistently, we found that serum UA levels were lower in patients after deferasirox treatment, providing clinical evidence for the UA-lowering effect of deferasirox (Fig. 2G–I).

In summary, our study successfully established cellular and animal models for screening XO inhibitors and identified deferasirox as a specific inhibitor of human XO. The cellular model provides a convenient and efficient platform for screening large compound libraries. We further validated the inhibitory effect of deferasirox on XO using human primary hepatocytes-derived liver organoids *in vitro* and a hyperuricemia animal model *in vivo.* Interestingly, deferasirox did not exhibit inhibitory effects on mouse XO, highlighting the importance of considering species differences when developing new XO inhibitors. To address this issue, we also developed a humanized animal model with human XO replacing the mouse XO and liver depletion of *Uox* to mimic human purine metabolism, enabling the evaluation of human XO inhibitors *in vivo* (Fig. 2J). Overall, our study identified deferasirox as a promising inhibitor of human XO, providing a foundation for the development of improved XO inhibitors for the treatment of gout or hyperuricemia. Importantly, our findings also highlight the crucial need to consider species differences when developing new XO inhibitors.

## Research limitations

The mechanism underlying the inhibition of human XO by deferasirox remains unclear. However, the selective inhibitory effect of deferasirox on human XO, but not mouse XO, suggests a direct binding interaction between deferasirox and human XO. Further investigations are required to elucidate the structural differences between species that may contribute to the binding of deferasirox to human XO. The efficacy tests conducted in animals demonstrated that deferasirox, while not as potent as allopurinol, may serve as a safer XO inhibitor. Analysis of patients regularly treated with deferasirox showed significant UA level reductions, confirming its UA-lowering effects [7–9]. However, some studies reported increased serum creatinine and decreased eGFR after 6 months, suggesting glomerular nephropathy [7]. Conversely, other studies found normal serum and creatinine clearance in treated patients [9]. Note that these human experiments were long-term. Clinical trials are needed to investigate short-term deferasirox treatment’s effectiveness and side effects in hyperuricemia patients. Moreover, deferasirox boasts high oral bioavailability and a relatively long half-life. Compared to allopurinol and febuxostat, deferasirox’s once-daily dosing regimen enhances patient compliance and convenience. Overall, deferasirox may offer an alternative therapeutic option for patients who are intolerant or unresponsive to current UA-lowering medications.

## Supplementary Material

lnaf014_suppl_Supplementary_Figures_S1-S2_Table_S1
