# Supplementary material for: Identification of deferasirox as a human xanthine oxidase inhibitor
Source: Life Med. 2025 Mar 31;4(2):lnaf014. doi: 10.1093/lifemedi/lnaf014 (PMC12075764; doi:10.1093/lifemedi/lnaf014)
Supplement: lnaf014_suppl_Supplementary_Figures_S1-S2_Table_S1 [file lnaf014_suppl_supplementary_figures_s1-s2_table_s1.docx]

**Identification of deferasirox as a human xanthine oxidase inhibitor**

Yunfei Qi^1^, Xinheng He^2^, Xiaoshan Wu^1^, Tingting Zhou^1^, Ningning Liang^1^, Jiazheng Jiao^1^, Yanhao Chen^1^, Yue Yuan^1^, Yuwei Zhang^1^, Yuchen Wang^1^, Yan Liu^1^, Qiurong Ding^1,#^

^1^CAS Key Laboratory of Nutrition, Metabolism and Food Safety, Shanghai Institute of Nutrition and Health, Shanghai Institutes for Biological Sciences, University of Chinese Academy of Sciences, Chinese Academy of Sciences, Shanghai 200031, China

^2^CAS Key Laboratory of Receptor Research, Shanghai Institute of Materia Medica, University of Chinese Academy of Sciences, Chinese Academy of Sciences, Shanghai 201203, China

^#^Correspondence: qrding@sinh.ac.cn (Q.D.)

**Supporting information**

Figures S1 and S2

Table S1

Methods

Data availability


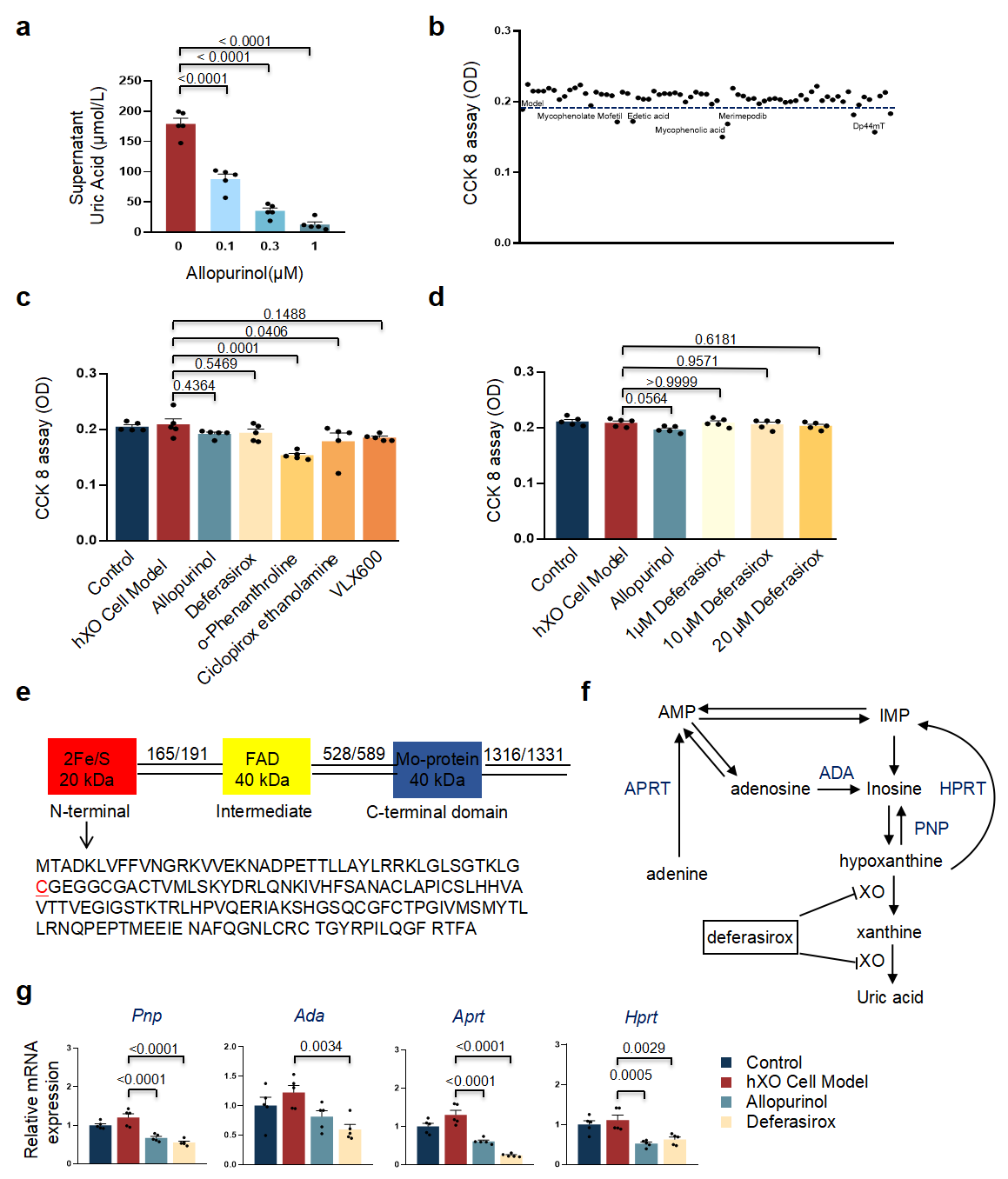


**Figure S1. High-throughput screening identifies deferasirox as an effective human xanthine oxidase inhibitor.** (A) Analysis of supernatant uric acid levels in Flag-hXO cells after treatment of allopurinol at different concentrations (0.1 μM, 0.3 μM, or 1 μM), as indicated. (*n* = 5). (B) Cell variability analysis after treatment of different compounds in high-throughput screenings. (C) Cell variability analysis after treatment with individual compound at a concentration of 10 μM, as indicated. *n* = 5. (D) Cell variability analysis after treatment with allopurinol (1 μM) or deferasirox at different concentrations, as indicated. *n* = 5. (E) Structure illustration of the XO protein, with the red C being C43. (F) Illustration of the steps and enzymes in purine metabolism. (G) Analysis of gene expression levels in cells after treatment with deferasirox (10 μM) or allopurinol (1 μM). *n* = 5. Values present means with s.e.m. *P* values were assessed by the one-way ANOVA (A, C, D and G).


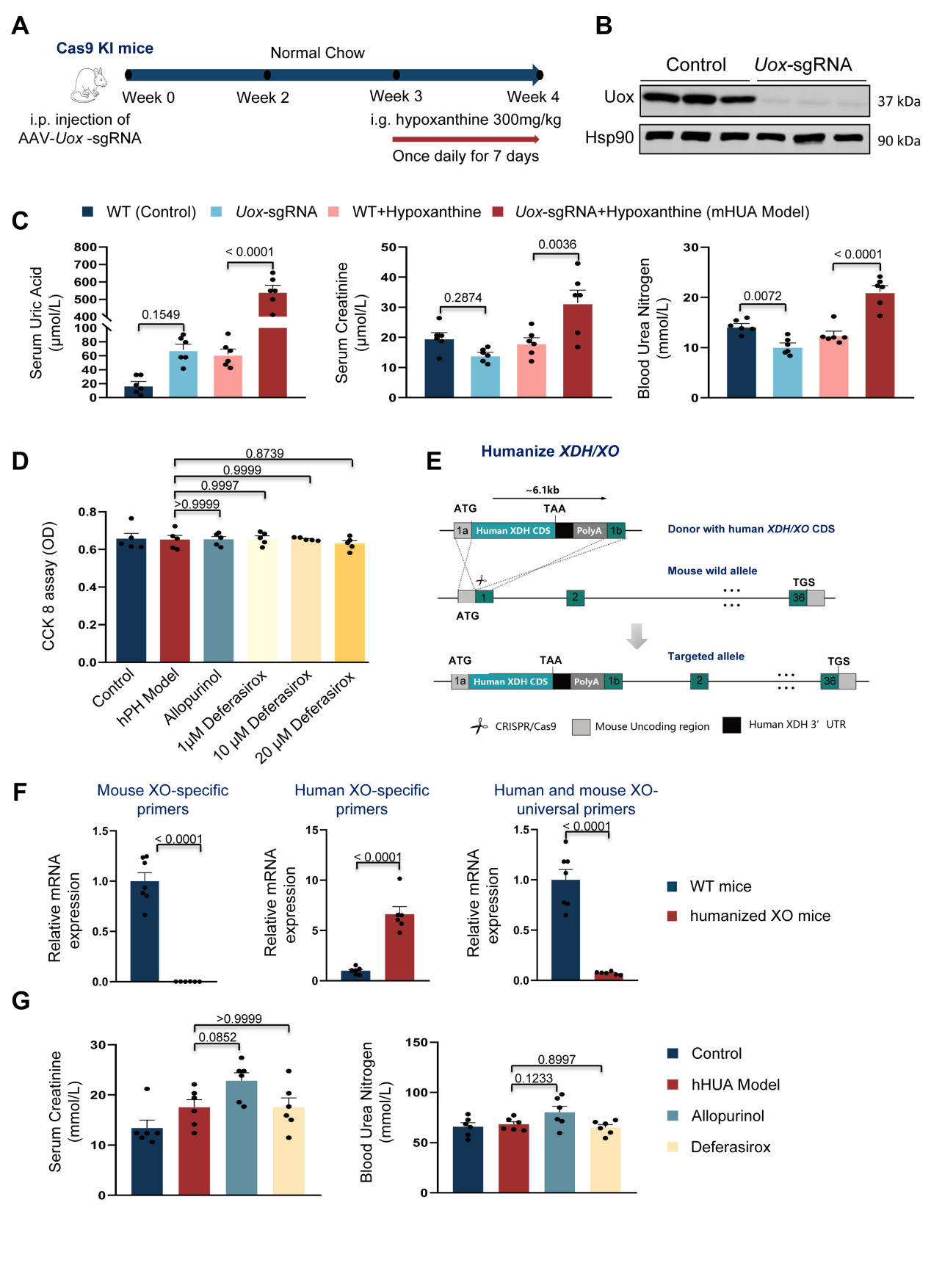


**Figure S2. Deferasirox has no inhibitory effect on mouse XO.** (A) Scheme illustration of establishing a mouse hyperuricemia model (mHUA model). (B) Western analysis of Uox protein expression in liver tissues after CRISPR/Cas9 targeting. (C) Analysis of serum uric acid, creatinine, and blood urea nitrogen in animals from different groups, as indicated. WT (Control), animals injected AAV vector were orally administrated with 0.5% CMC-Na solution; *Uox*-sgRNA, animals injected AAV-*Uox*-sgRNA were orally administrated with 0.5% CMC-Na solution; WT + hypoxanthine, animals injected AAV vector were orally administrated with 300 mg/kg hypoxanthine; *Uox*-sgRNA + hypoxanthine (mHUA model), animals injected AAV-*Uox*-sgRNA were orally administrated with 300 mg/kg hypoxanthine. *n* = 6. (D) Cell viability analysis of hPH Models after treatment with deferasirox at different concentrations, as indicated. Allopurinol (1 μM) was applied as a positive control. *n* = 5. (E) Scheme illustration of generating humanized animals with human XO replacing mouse XO. (F) Analysis of mouse XO or human XO mRNA expression levels in liver tissues from the wild-type and humanized animals, respectively. *n* = 6 or 7. (G) Analysis of serum creatinine and blood urea nitrogen in animals from different groups, as indicated. *n* = 6. Values present means with s.e.m. *P* values were assessed by the one-way ANOVA (C, D and G), or the unpaired *t*-test (F).

**Table S1. List of compounds in the metal chelators library**

| Molecular name | Cas |
| --- | --- |
| Etidronate | 2809-21-4 |
| Dithiopropanol | 59-52-9 |
| Pentetic Acid | 67-43-6 |
| Hydroxyquinoline | 148-24-3 |
| Lithium citrate tribasic tetrahydrate | 6080-58-6 |
| Sodium salicylate | 54-21-7 |
| Succinic acid | 110-15-6 |
| d-penicillamin | 52-67-5 |
| Ciclopirox ethanolamine | 41621-49-2 |
| Formononetin | 485-72-3 |
| Clioquinol | 130-26-7 |
| Salicylic acid | 69-72-7 |
| Tannic acid | 1401-55-4 |
| Delta-Gluconolactone | 90-80-2 |
| Deferasirox | 201530-41-8 |
| Cisplatin | 15663-27-1 |
| Deferidone | 30652-11-0 |
| Deferoxamine Mesylate | 138-14-7 |
| Silibinin | 22888-70-6 |
| Ipragliflozin | 761423-87-4 |
| Molecular name | Cas |
| Succimer | 304-55-2 |
| Kaempferol | 520-18-3 |
| Daidzin | 552-66-9 |
| Methylcobalamin | 13422-55-4 |
| Pyrrolidinedithiocarbamate ammonium | 5108-96-3 |
| Mycophenolic acid | 24280-93-1 |
| Empagliflozin | 864070-44-0 |
| Dapagliflozin | 461432-26-8 |
| Inauhzin | 309271-94-1 |
| BAPTA-AM | 126150-97-8 |
| Verbascoside | 61276-17-3 |
| Sotagliflozin | 1018899-04-1 |
| Mycophenolate Mofetil | 128794-94-5 |
| L-Mimosine | 500-44-7 |
| Galangin | 548-83-4 |
| Merimepodib | 198821-22-6 |
| TPEN | 16858-02-9 |
| Lutidinic acid | 499-80-9 |
| Citric acid | 77-92-9 |
| Dp44mT | 152095-12-0 |
| Maleic acid | 110-16-7 |
| Molecular name | Cas |
| L-Lactic acid | 79-33-4 |
| UDP disodium salt | 27821-45-0 |
| 2,3-Dihydroxybenzoic acid | 303-38-8 |
| L(+)-Tartaric acid | 87-69-4 |
| Adenosine-5'-diphosphate trisodium salt | 20398-34-9 |
| Ertugliflozin | 1210344-57-2 |
| (L)-Sodium lactate | 867-56-1 |
| o-Phenanthroline | 66-71-7 |
| Potassium acetate | 127-08-2 |
| Tropolone | 533-75-5 |
| VLX600 | 327031-55-0 |
| Edetic acid | 60-00-4 |
| KGA-2727 | 666842-36-0 |
| Mizagliflozin | 666843-10-3 |
| Imidazole | 288-32-4 |
| Phytic acid | 83-86-3 |
| Mizoribine | 50924-49-7 |
| Morin | 480-16-0 |
| Dexrazoxane HCl | 149003-01-0 |
| Trientine-2HCl | 38260-01-4 |
| Rhodamine 6G | 989-38-8 |
| Molecular name | Cas |
| Miriplatin | 141977-79-9 |
| Clodronate Disodium | 22560-50-5 |
| Sodium gluconate | 527-07-1 |
| L-Methionine | 63-68-3 |
| Inosine 5'-triphosphate trisodium salt | 35908-31-7 |
| Guanosine 5'-triphosphate trisodium salt | 36051-31-7 |
| Sodium phytate hydrate | 14306-25-3 |
| Deferitazole | 945635-15-4 |

**Methods**

**Cell culture**

AML12 (alpha mouse liver 12) cells (Cell Bank, Type Culture Collection Committee, Chinese Academy of Sciences) were maintained in DMEM/F-12 (ThermoFisher, C11330500CP) containing 10% FBS (Gibco, 16000044), 1% ITS (Sigma, I3146), 40 ng/ml dexamethasone (Sigma, D4902) and 1% penicillin–streptomycin mixture (YEASEN, 60162ES76) at 37°C under 5% CO_2_.

For generation of stable AML12 cell lines with exogenous hXO or mXO expression, hXO cDNA (encoding human xanthine oxidase) was cloned from the pcDNA3.1mychisA XDH plasmid (Addgene, 10958), and inserted into the lentiviral expression vector pCDH-EF1-3×Flag-MCS-T2A-puro between the NotⅠ and XbaⅠ sites. AML12 cells stably overexpressing hXO or empty vector were selected for one week by 2 ug/mL puromycin. The efficacy of stable hXO overexpression was confirmed by qPCR analysis and Western blotting. The hXO-C43A mutant plasmid was constructed by replacing the 43 cysteine to alanine via the homologous recombination approach using hXO as template. mXO cDNA (encoding mouse xanthine oxidase) was cloned from mouse cDNA by PCR. Similar approaches were adopted via lentiviral delivery and puromycin selection for generation of stable cell lines expressing hXO-C43A and mXO, respectively.

**Establishment of the hyperuricemia cell model for high-throughput drug screening**

The hXO stable AML12 cells were cultured in 96-well plates (Corning, 4442) (2×10^4^ cells/well) and grown for 24 h, followed by PBS wash and supplementation of guanosine (Sigma, G6752) and inosine (Sigma, I4125) (100 μM each) for 8 h in serum-free, phenol red-free DMEM medium (Pricella, PM150223). Analysis of UA levels in supernatant was then performed using the uric acid test kit (Nanjing Jiancheng Bioengineering Institute, C012-2-1).

For exploration of time-dependent UA production, cells were exposed to guanosine and inosine (100 μM each) for 2, 4, 6, 8, 10, and 12 h, respectively, before 50 μL supernatant was collected for UA level determination.

For observation of the UA-lowering effect by allopurinol as a positive control, allopurinol (Sigma, A8003) was applied together with guanosine and inosine at different concentrations (0.1 μM, 0.3 μM, or 1 μM) for 8 h before UA analysis.

For high-throughput screening of metal chelators, a compound library containing 70 compounds previously being reported to have metal chelating activity in the literature were compiled (TargetMol, LC00). Cells were pretreated with 10 μM of individual compound for 24 h, followed by supplementation of guanosine and inosine for 8 hours and UA determination. Cell viability was detected using Cell Counting Kit-8 (TarketMol, C0005).

After high-throughput drug screening, 10 μM Deferasirox (TargetMol, T1457), o-Phenanthroline (TargetMol, T5983), Ciclopirox ethanolamine (TargetMol, T1114), and VLX600 (TargetMol, T8500) were further individually confirmed for its impact on UA production as well as cell activity.

**Functional evaluation of deferasirox using human primary hepatocyte-derived liver organoids**

Human primary hepatocytes (kindly provided by Dr. Hui Lijian, Center for Excellence and Innovation in Molecular Cell Science, Chinese Academy of Sciences) were cultured following an *in vitro* expansion and maturation protocol as described previously (6). Briefly, the primary human hepatocytes were seeded at 3 × 10^4^ cells/cm^2^ and passaged in every 5–6 days in 6-well collagen-I-coated plates (Corning, 4442) in HM medium for expansion. For hepatic maturation, cells were seeded at 8 × 10^4^ cells/well in a non-adherent surface 96 well plate (Kuraray, RB 500 400 NA 96) in HIM medium and grown as liver organoids in a normoxia incubator (5% CO_2_, 37°C) for 10 days. For testing the efficacy of deferasirox, 10 μM deferasirox, or 1 μM allopurinol as positive control, or DMSO as vehicle control, was added to the medium and incubated for 24 h at Day 11. Then the medium was replaced with serum-free, phenol red-free DMEM medium with guanosine and inosine (100 μM each) supplemented for 8 h, followed by supernatant collection and determination of UA production using Q Exactive PLUS hybrid quadrupole–orbitrap mass spectrometer (Thermo Scientific).

**Animals**

All animals were maintained and used in accordance with the guidelines of, and under the approval by the Institutional Animal Care and Use Committee of the Shanghai Institute for Nutrition and Health (ethical committee approval No. SINH-2020-DQR-3). The animals presented a healthy status and male mice were used for all experiments.

The colony of Cre-dependent *Cas9* knockin mice (Rosa*Cas9*^+/−^) (The Jackson Laboratory) was maintained by crossing with the wild-type C57BL/6J mice (Shanghai Laboratory Animal Center). For liver-specific depletion of *Uox*, 8-week-old male homozygous *Cas9* knockin animals were randomly divided into groups, and adeno-associated virus 8 (AAV8) vectors expressing Cre recombinase and *Uox*-sgRNA (GTTCTCCATATTCAGAGAGA+AGG) were administered by tail vein injection. A firefly luciferase expression cassette was also included in the AAV vector to assist *in vivo* evaluation of delivery efficiency. AAV vectors with Cre recombinase and luciferase cassettes and no sgRNA were used as control viruses. Viruses were dissolved in 200 μL phosphate-buffered saline (PBS) and were administered at a dose of 2 × 10^11^ vector genomes (vg) per mouse. Three weeks after virus injection, the knockout efficiency by CRISPR targeting in liver tissues was examined with the uricase antibody (Santa Cruz Biotechnology, sc-166214) by Western blotting.

For establishing an animal model with hyperuricemia, three weeks after AAV injection for liver *Uox* depletion, animals were orally administrated with hypoxanthine (300 mg/kg) (MCE, HY-N0091) once daily for 7 days, or 0.5% CMC-Na (MCE, HY-Y1889A) solution as vehicle control. For drug treatment, 20 mg/kg deferasirox or allopurinol (as positive control) were orally administrated once daily 2 h after hypoxanthine administration.

The humanized XO animals were generated in GemPharmatech, in which the human *XO/XDH* CDS and part of the 3'UTR region was inserted after the ATG start codon of mouse XO gene with the addition of a PolyA termination signal, resulting in the termination of mouse XO expression. The humanized XO mice were then crossed with the *Cas9* knockin animals for further *Uox* depletion via AAV delivery of sgRNAs targeting *Uox*, followed by hypoxanthine administration and drug treatment as described above.

For measurement of physiological parameters, the uric acid test kit (Nanjing Jiancheng Bioengineering Institute, C012-2-1), creatinine assay kit (Nanjing Jiancheng Bioengineering Institute, C011-2-1), and blood urea nitrogen assay kit (Nanjing Jiancheng Bioengineering Institute, C013-1-1) were adopted to measure serum UA, creatinine, and blood urea nitrogen levels, respectively. In humanized XO mice, due to relatively low levels, serum uric acid, creatinine and blood urea nitrogen were determined using a clinical chemistry analyzer DRI-CHEM NX700V (FUJIFILM).

**RNA isolation and RT-qPCR analysis**

Total RNA was extracted from liver tissues by using TRIzol reagent (Thermo Fisher Scientific, 15596018) according to the manufacturer’s instructions. Reverse transcription of isolated RNA was performed using the HiScript II Reverse Transcriptase (Vazyme, R201-01). Quantitative real-time PCR was carried out on the 7900 System (Applied Biosystems, 4472908) using Taq Pro Universal SYBR qPCR Master Mix (Vazyme, Q712-02/03). The sequences of primers used are as follows:

Mouse *18s* forward: 5'-AAACGGCTACCACATCCAAG-3',

Mouse *18s* reverse: 5'-CCTCCAATGGATCCTCGTTA-3';

Human *XO* forward: 5'-CCAAATTGCTGCATGAACCAG-3',

Human *XO* reverse: 5′-TGCTTCCGAGGAGTGTCTTTC-3′;

Mouse *XO* forward: 5'-ATGACGAGGACAACGGTAGAT-3',

Mouse *XO* reverse: 5'-TCATACTTGGAGATCATCACGGT-3';

Human/mouse *XO* forward: 5'-GAGAGAAGATGACATTGCCA-3',

Human/mouse *XO* reverse: 5'-GAGTGGTCTTGAGGGCTGAG-3';

Mouse *Pnp* forward: 5'-CAACACACTGAATATCGACCTCA-3',

Mouse *Pnp* reverse: 5'-GCTTTGGGGAAAGTTGGGTATCT-3';

Mouse *Ada* forward: 5'-ACCCGCATTCAACAAACCCA-3',

Mouse *Ada* reverse: 5'-AGGGCGATGCCTCTCTTCT-3';

Mouse *Aprt* forward: 5'-CCCTCTTGAAAGACCCGGAC-3',

Mouse *Aprt* reverse: 5'-TCCAGAGAATAGGAGGCTGAC-3';

Mouse *Hprt* forward: 5'-TCAGTCAACGGGGGACATAAA-3',

Mouse *Hprt* reverse: 5'-GGGGCTGTACTGCTTAACCAG-3';

Mouse *Havcr1* forward: 5'-ACATATCGTGGAATCACAACGAC-3',

Mouse *Havcr1* reverse: 5'-ACAAGCAGAAGATGGGCATTG-3';

Mouse *Tnfa* forward: 5'-CCCTCACACTCAGATCATCTTCT-3',

Mouse *Tnfa* reverse: 5'-GCTACGACGTGGGCTACAG-3';

Mouse *Il6* forward: 5'-TAGTCCTTCCTACCCCAATTTCC-3',

Mouse *Il6* reverse: 5'-TTGGTCCTTAGCCACTCCTTC-3';

Mouse *Nlrp3* forward: 5'-ATCAACAGGCGAGACCTCTG-3',

Mouse *Nlrp3* reverse: 5'-GTCCTCCTGGCATACCATAGA-3'.

**Western blotting**

Protein from cells or tissues was extracted using RIPA buffer (Millipore, 20188) and was subjected to western blotting. The primary antibodies used in the experiments were antibodies to FLAG (Sigma-Aldrich, F7425), GAPDH (Proteintech, 60004-1), Hsp90 (Proteintech, 60318-1-Ig) and Uox (Santa Cruz Biotechnology, sc-166214).

**Statistics analysis**

Statistic differences between different groups were analyzed using the unpaired, two-tailed Student’s *t* test, One-way ANOVA, or Two-way ANOVA as indicated in the figure legends. Statistical significance was determined as P < 0.05, with the exact P values, or 0.0001 when the P value was equal or less than 0.0001, displayed in the figures. P values were calculated in GraphPad Prism 8.0. All data were presented as means with s.e.m.

**Data availability**

No dataset has been generated in this study. Other data obtained and analyzed during this study are available from the corresponding author once reasonably requested.
